# Associations of body mass index and waist circumference with: energy intake and percentage energy from macronutrients, in a cohort of australian children

**DOI:** 10.1186/1475-2891-10-58

**Published:** 2011-05-26

**Authors:** Sarah A Elliott, Helen Truby, Amanda Lee, Catherine Harper, Rebecca A Abbott, Peter SW Davies

**Affiliations:** 1 The University of Queensland, Children's Nutrition Research Centre, School of Medicine, Royal Children's Hospital, Brisbane, Australia; 2 Monash University, Southern Clinical School, Department of Nutrition and Dietetics, Clayton VIC 3168, Australia; 3 Nutrition and Physical Activity Health Promotion Branch, Queensland Health, Brisbane, QLD 4001, Australia; 4 Planning and Development Unit, Population Health Queensland, Queensland Health, Brisbane, QLD 4001, Australia; 5 School of Human Movement Studies, University of Queensland, Brisbane, QLD, 4069, Australia

**Keywords:** BMI, waist circumference, macronutrient intake, energy intake, children, obesity

## Abstract

**Background:**

It is evident from previous research that the role of dietary composition in relation to the development of childhood obesity remains inconclusive. Several studies investigating the relationship between body mass index (BMI), waist circumference (WC) and/or skin fold measurements with energy intake have suggested that the macronutrient composition of the diet (protein, carbohydrate, fat) may play an important contributing role to obesity in childhood as it does in adults. This study investigated the possible relationship between BMI and WC with energy intake and percentage energy intake from macronutrients in Australian children and adolescents.

**Methods:**

Height, weight and WC measurements, along with 24 h food and drink records (FDR) intake data were collected from 2460 boys and girls aged 5-17 years living in the state of Queensland, Australia.

**Results:**

Statistically significant, yet weak correlations between BMI z-score and WC with total energy intake were observed in grades 1, 5 and 10, with only 55% of subjects having a physiologically plausible 24 hr FDR. Using Pearson correlations to examine the relationship between BMI and WC with energy intake and percentage macronutrient intake, no significant correlations were observed between BMI z-score or WC and percentage energy intake from protein, carbohydrate or fat. One way ANOVAs showed that although those with a higher BMI z-score or WC consumed significantly more energy than their lean counterparts.

**Conclusion:**

No evidence of an association between percentage macronutrient intake and BMI or WC was found. Evidently, more robust longitudinal studies are needed to elucidate the relationship linking obesity and dietary intake.

## Background

Several studies investigating the relationship between body mass index (BMI), waist circumference (WC) and/or skin fold measurements with energy intake have suggested that the macronutrient composition of the diet (protein, carbohydrate, fat) may play an important contributing role to obesity in childhood as it does in adults [1-4]. A report by the World Health Organisation in 2000 revealed that worldwide over 1.5 billion adults are overweight, with approximately 4 million of these adults being classified as clinically obese [5]. According to Rodriguez *et al*. (2006) the proportion of children who are overweight or obese ranges between 15-25% in many Western societies [6]. Equally alarming, however, is the dramatic increase in the prevalence of childhood obesity which, in Australia, has more than doubled within the last two decades [7,8].

The Australia Health and Fitness Survey conducted in 1985, reported that 10.7% of boys and 11.8% of girls aged seven to fifteen years were overweight or obese [9]. Margery *et al*. (2001) reanalysed data from this study and the 1995 Australian National Nutrition Survey [10] using Body Mass Index (BMI) cut-off points established by the International Obesity Task Force [11], and concluded that the prevalence of overweight and obese children aged 7 to fifteen years had increased to 20% for boys and 21.5% for girls [12].

Since 1995, in Australia, there have been no nationally representative studies into the habitual nutritional intakes of children and the population of children who are overweight and/or obese. Three individual states (New South Wales, Western Australia, and Queensland) however, have recently conducted state based surveys [13,14] illustrating that the number of overweight and obese children had increased in recent years.

The aetiology of obesity, like most biological functions, is multi-factorial but is believed to arise due to an imbalance in the generalised energy balance equation [15,16]. However, some studies, which have investigated energy intake in children, have failed to show that fatter children have higher reported energy intakes, which may have been due to underreporting of food intake in obese children/adolescents [17,18]. Statistical techniques to reduce reporting bias which exclude illogical or invalid (either from over or underreporting) energy intake data, have been developed by Goldberg *et al*. (1991) and further refined by McCrory *et al*. (2002) [19,20].

Studies investigating the relationship between energy intake and adiposity in children and adolescents have produced confounding results. While some studies have found an inverse relationship, others have found no association between the two variables. As a result of the discrepancies between studies and the inconsistency of results, researchers have started to explore the possible relationship between adiposity and diet composition (percentage energy intake from macronutrients). Several cross sectional studies have reported a positive relationship between fat intake and the degree of adiposity in children [3,18,21] while others have not [12,22]. There are only few published studies, however, which have specifically addressed fat, protein and carbohydrate intake in relation to BMI and WC in children [23,24]. From these studies it is evident that the role of dietary composition in relation to the development of childhood obesity remains inconclusive and requires more attention.

The aim of the present study was to explore the possible relationships of BMI and WC in children and adolescents and relate these parameters to gross daily energy intake and the percentage energy intake derived from macronutrients. As under-reporting of energy intake is common, particularly in overweight populations [17,25,26] the effect of mis-reporting on these associations was addressed at the outset by applying the McCrory cut-off [20] which eliminated both under and over reporters of energy intake.

## Methods

### Subjects

The Healthy Kids Queensland Survey took place throughout Queensland, Australia from April to September 2006. A random sample of 112 schools from all primary and secondary schools taken from government and non-government sectors were invited to take part. A random cluster design was used and the data were weighted to ensure equal probability of inclusion of all children in the target population. Further, to maximise the statistical power of the survey, three key age groups at critical times in growth and development were chosen: the first year of compulsory schooling, i.e. about 5 years of age (Year 1), just prior to puberty, i.e. about 9-11 years of age (Year 5) and about 14 to 16 years of age, (the last year of compulsory schooling), (Year 10).

The survey aimed to recruit children representative of Queensland children and, to this end, 59 schools in urban areas and 53 from rural areas were chosen randomly. Of these, 72 agreed to take part. The sample represented a mix of 39 schools in urban areas and 33 schools in rural areas. The definition of an urban school was that the school was based in a location with an Accessibility-Remoteness Index of Australia Plus (ARIA+) category of 1 and was deemed highly accessible (AIHW, 2004). A rural school was defined as a school location with an ARIA+ category of 2-4 that was deemed accessible through to remote. The only exclusion criteria were schools with fewer than 25 students, special schools and schools that were classified as 'very remote' according to ARIA+. A total of 3691 children and adolescents from years 1, 5 and 10 participated in the survey. The Healthy Kids Queensland 2006 survey was conducted according to the guidelines laid down in the Declaration of Helsinki and all procedures involving human subjects were approved by The University of Queensland Ethics Committee, and the Education Queensland Ethics Committee. Informed written consent was obtained from both the participating subject and their parent/guardian before the study commenced.

### Anthropometric Measurements

Height and weight were measured and data were collected as described by Davies *et al.*, 200[27]. Height was measured twice to the last completed millimetre and the mean of the two measurements recorded. If the two measurements differed by more than 5 mm, height was recorded again until two measurements which did not differ by more than 5 mm were recorded. Body weight was measured once, to the nearest 0.1 kg using digital scales (Tanita HD316, Tokyo, Japan). WC was measured to the nearest 0.1 cm in two places: around the umbilicus, and half way between the last rib and iliac crest. The mean of these two measures were included in the analysis. BMI was calculated using height and weight, and converted into age and gender specific BMI z-scores using LMS reference parameters provided by the Centres for Disease Control (CDC Growth Charts, Accessed 9 March 2007) to provide a continuous variable, appropriate for comparing overweight and obesity among children [28]. However, raw WC data split into age and sex bands were used instead of z-scores as previous studies have shown that there is a relationship between age and WC [29].

### 24 hr Food and Drink Record

Food and drink records over a 24-hour period (24 hr FDR) were completed by all Grade 5 and 10 children and 25% of Grade 1 children. A comprehensive 24 hr FDRs methodology is detailed in the Healthy Kids Queensland Survey 2006 Summary Report [30]. In summary, children were shown how to complete a 24 hr FDR in a classroom presentation. They were asked to record information on the day/date the record was completed, start (time they woke-up) and finish (bedtime) times, detailed descriptions of all foods and beverages consumed during the period they were awake, recipes, as well as the amounts eaten. They were provided with a standard set of measuring cups and spoons and a ruler to assist with quantification of foods and beverages consumed. Parents, of children in Year 1 were asked to assist the children in completing the records if necessary. All 24 hr FDRs were reviewed by the research team with each individual student (and parents of the youngest children), to ensure sufficient detail and accuracy of records and measurement were provided for the analysis of dietary intake.

The 24 hr FDRs were analysed using FoodWorks Professional Edition (2005 Version 4.00) Foodworks is a nutrition analysis software program that uses the AUSNUT database (produced by Food Standards Australia New Zealand). For energy intake analysis, Foodworks output presented data on total energy intake as well as energy from protein; carbohydrates and fat (saturated, polyunsaturated, monounsaturated).

A team of coders completed the 24 hr FDR data entry. Two team members were responsible for researching, developing coding instructions, and checking queries for quality control purposes and to ensure consistency between all the coders. All entered data were then assessed twice by two different coders to eliminate errors that would affect total energy intake calculations.

### Identification of mis-reporters of energy intake

Basal Metabolic Rate (BMR) was predicted using age and gender specific equations, taking into account measured height and weight [31]. Total Energy Expenditure (TEE) was estimated from BMR multiplied by a physical activity level, assumed to be 1.5 (PAL = 1.5) [32]. A ratio of reported energy intake to predicted TEE was calculated for each child as suggested by McCrory et al. (2002) [20]. The plausible range of these ratios was calculated using data from Huang *et al*. (2004) [32]. The equation used takes into account the coefficient of variation in energy intake reporting, the coefficient of variation in predicting total energy expenditure using published equations and the coefficient of variation in total energy expenditure as measured by doubly labelled water. Only those who returned physiologically plausible energy intake records have been used in this analysis.

### Statistical Analysis

A detailed description of the sampling method has been reported in the HKQ Summary report. All data arising from the survey was weighted to ensure the equal probability of inclusions of all children in the target population. Results are reported as mean ± standard deviation. Associations between dietary intake variables and BMI z-score and WC were examined by Pearson's product-moment correlation coefficients. To see if there was any association between energy intake and macronutrient intake with BMI and WC, subjects were divided into quartiles based on their BMI z-score and WC in centimetres. Mean energy intakes and percentage energy from various macronutrients were ascertained for each quartile. Dunnets post hoc tests were performed to examine which quartiles were significantly different to one another.

Regression analysis was used to determine the extent to which diet composition, age and gender were associated with BMI and WC. Statistical analyses were performed using the SPSS for Windows statistical package (Version 17.0; SPPS Inc, Chicago, IL.).

## Results

Data were collected throughout Queensland from April to September 2006. The HKQ survey aimed to recruit children across Queensland, and to this end, 59 schools in urban areas and 53 from rural areas were chosen randomly. Of these, 72 agreed to take part - a response rate of 65%. The sample represented a mix of 39 schools in urban areas and 33 schools in rural areas. Of the 3691 children who participated in the survey 2460 subjects had both complete 24HFDR and anthropometric data (Height, Weight, and WC). The participation rates in this aspect of the survey were 85%, 94% and 92% in grades 1, 5, and 10, respectively.

After screening out "implausible" energy intake records using the McCrory approach, 1352 records (55%) remained (Table 1). The mean and standard deviation for energy intake and percentage energy intake from various macronutrients for both sexes in the 3 year groups are shown in Table 1. There was a difference in energy intake between the two sexes in all age groups, with males having a significantly higher energy intake than females. Macronutrient distribution on average was protein 17%, carbohydrate 51%, and fat 32% of energy intake.

**Table 1:** Subject characteristics, energy intake and percentage macronutrient intake in plausible energy reporters (n = 1352)

	n	Age (years)	Height (cm)	Weight (kg)	BMI z-score	WC (cm)	Energy Intake (kj/day)	Fat	Carbohydrate	Protein
Year 1										
Male	59	6.2 ± 0.36	119.8 ± 5.9	24.1 ± 5.6	0.7 ± 1.1	58.4 ± 7.0	^1^6954 ± 9291	31.5 ± 5.7	52.6 ± 6.5	15.9 ± 3.3
Female	68	6.1 ± 0.38	116.9 ± 4.7	21.9 ± 3.4	0.3 ± 0.8	55.8 ± 4.6	6197 ± 643	32.3 ± 5.8	51.6 ± 7.0	16.1 ± 3.4
Year 5										
Male	333	10.2 ± 0.4	141.0 ± 6.5	36.1 ± 7.7	0.3 ± 1.0	65.5 ± 8.8	^1^8289 ± 14681	32.9 ± 5.8	50.6 ± 7.5	16.4 ± 4.4
Female	424	10.1 ± 0.4	141.5 ± 6.6	37.5 ± 8.6	0.4 ± 1.0	66.8 ± 9.5	7455 ± 1204	33.0 ± 6.4	50.5 ± 7.9	16.5 ± 4.4
Year 10										
Male	181	15.2 ± 0.4	172.5 ± 8.7	63.3 ± 11.7	0.2 ± 1.0	76.5 ± 7.6	^1^11165 ± 22111	32.9 ± 7.0	49.7 ± 8.8	17.3 ± 4.8
Female	287	15.2 ± 0.4	163.8 ± 5.7	56.5 ± 9.5	0.1 ± 0.9	74.6 ± 7.4	8446 ± 1485	33.0 ± 7.7	49.8 ± 8.9	16.9 ± 4.9

Values are means ± standard deviation. WC; waist circumference. Fat, Carbohydrate and Protein are expressed as % of energy intake.

^1^Significantly different to female

BMI z-score was significantly but modestly correlated with total energy intake across all age groups (Table 2). However, no correlation was found between BMI z-score and percentage energy intake from protein, carbohydrate or fat. Likewise, there was a significant correlation between WC and energy intake across all age groups (Table 2).

**Table 2:** Pearson product-moment correlation coefficients between BMI z-score and WC with dietary intake variables

		Year 1	Year 5	Year 10
		n = 127	n = 757	n = 468
		r	r	r
BMI				
	Energy (kj/day)	0.26*	0.39*	0.23*
	Fat (% of energy)	-0.05	-0.03	-0.03
	Carbohydrate (% of energy)	0.01	0.02	-0.01
	Protein (% of energy)	0.06	0.01	0.05
WC				
	Energy (kj/day)	0.32*	0.43*	0.31*
	Fat (% of energy)	-0.15	0.01	-0.02
	Carbohydrate (% of energy)	0.11	-0.01	-0.02
	Protein (% of energy)	0.04	0.01	0.05

* p < 0.01

When subjects were grouped based on quartiles of BMI z-score, a one way ANOVA showed that those with a higher BMI z-score consumed significantly more energy than their lean counterparts, however, percentage of protein, carbohydrate and fat intake were similar across the groups (Table 3). When grouping subjects based on quartiles of WC, there was no significant difference between quartiles in any of the age groups for any macronutrient (Table 4). For year 1 children there was no significant difference in total energy intake across the quartiles of WC, however in years 5 and 10, there was a significant increase in total energy intake in relation to WC (Table 4).

**Table 3:** Energy intake and percentage energy from macronutrients based on quartiles of BMI z-score

	Quartile 1	Quartile 2	Quartile 3	Quartile 4
	n = 338	n = 338	n = 338	n = 338
BMI z-score	-0.9 ± 0.6	-0.01 ± 0.2	0.63 ± 0.2	1.47 ± 0.5
Total Energy	7837 ± 1742^1,2^	8089 ± 1853^2^	8306 ± 1758^2^	8896 ± 240
FAT (% of energy)	33.6 ± 7.1	32.7 ± 6.7	32.8 ± 5.9	32.7 ± 6.6
CHO (% of energy)	50.0 ± 8.6	50.9 ± 8.1	50.4 ± 7.5	50.4 ± 7.9
PRO (% of energy)	16.5 ± 4.6	16.4 ± 4.3	16.8 ± 4.4	16.8 ± 4.6

Values are means ± standard deviation.

1 Significantly different to quartile 3

2 Significantly different to quartile 4

(p < 0.05) ANOVA

**Table 4:** Energy intakes and % energy from macronutrients based on quartiles of waist circumference in year 1, 5 and 10.

	Quartile	N	WC (cm)	Total Energy (Kj/day)	% FAT	% CHO	% PRO
Year 1							
	1	32	51.3 ± 1.6	6337 ± 759	32.5 ± 6.1	51.8 ± 6.1	15.7 ± 2.8
	2	32	54.5 ± 0.8	6321 ± 844	33.5 ± 5.4	49.8 ± 6.3	16.8 ± 3.4
	3	32	57.5 ± 1.0	6703 ± 835	30.5 ± 6.2	53.9 ± 7.7	15.7 ± 3.2
	4	31	64.1 ± 6.2	6842 ± 961	31.4 ± 4.8	52.9 ± 6.5	15.8 ± 4.0
Year 5							
	1	190	56.7 ± 2.5	7145 ± 1080^1^	32.9 ± 6.6	50.7 ± 86	16.4 ± 4.7
	2	190	62.0 ± 1.4	7538 ± 1086^1^	33.0 ± 5.8	50.8 ± 7.4	16.3 ± 4.1
	3	188	67.2 ± 1.9	8084 ± 1265	33.4 ± 5.5	50.1 ± 7.0	16.5 ± 4.5
	4	189	79.1 ± 7.0	8527 ± 1641	32.6 ± 6.6	50.6 ± 7.8	16.7 ± 4.5
Year 10							
	1	117	66.8 ± 2.7	8600 ± 1682^1^	33.4 ± 7.8	49.5 ± 9.1	17.1 ± 5.1
	2	117	72.4 ± 1.3	9233 ± 1792^1^	33.6 ± 7.6	50.2 ± 8.5	16.3 ± 4.2
	3	117	76.8 ± 1.3	10007 ± 2299	32.9 ± 7.6	49.7 ± 9.2	17.4 ± 5.0
	4	117	85.2 ± 6.1	10151 ± 2687	32.7 ± 6.9	49.7 ± 8.6	17.4 ± 4.9

Values are means ± standard deviation

^1 ^Significant difference between quartiles of energy intake- significantly different to quartiles 3 and 4.

(p < 0.05)

Using multiple regression techniques, with BMI z-score as the independent variable, and percentage energy from protein, carbohydrate and fat as dependent variables (all entered together), neither the percentage carbohydrate, protein nor fat intake had a significant impact on predicting BMI z-score. Moreover, when using percentage energy from protein, carbohydrate and fat intake to predict WC in a multiple regression, none of the macronutrients had a significant affect.

## Discussion

Numerous studies in adults have found that diet composition may play an important role in the development of adiposity [2,4]. Researchers are now investigating the relationship between diet composition and adiposity in children [3,33]. Several studies investigating the relationship between BMI, waist circumference (WC) and/or skin fold measurements with energy intake have suggested that the macronutrient composition of the diet (protein, carbohydrate, fat) may play an important contributing role to obesity in childhood [33-35] as it does in adults [1-4]. However conflicting results have been found [18]. This study aimed to explore the possible relationship between BMI and WC with energy intake and percentage energy intake from macronutrients in children and adolescents, using a more robust technique by eliminating potential food reporting errors.

Studies using BMI as a parameter for "overweight" and "obesity" such as Rocandio *et al*., (2001) and Hassapidou *et al*., (2006) found that overweight adolescences of both sexes reported lower energy intake than non- overweight subjects [36,37]. However, our findings, where we have accounted for mis-reporters, demonstrate a weak but significant positive correlation between BMI z-score and energy intake. When examining the relationship between BMI z-score and energy intake based on quartiles, there was a significant difference amongst the groups, suggesting that our population of overweight children and adolescents consume more energy than their leaner counterparts. Similar findings were seen when using WC to assess the degree of overweight and obesity. Once again a weak but significant positive correlation between WC and total energy intake across all age bands was demonstrated. When comparing groups based on quartiles of WC there was significant difference between the groups in year five and ten. Yet, no significant differences between the groups were found in younger children (year 1).

Energy intake from carbohydrate has been shown in adults to be inversely associated with body fat [22,36,38]. Studies by Hassapidou *et al*. (2006) and Ortega *et al*. (1995) found that overweight and obese adolescents consumed fewer carbohydrates than lean subjects [23,37]. Again, once under-reporters are omitted, the results from this study differ in that there was no association between carbohydrate intake and either BMI z-score or WC. Again, there was no significant difference between the groups when comparing percentage carbohydrate intake based on quartiles of BMI z-score and WC. Similar to studies by Rocandio *et al*. (2001) and Maffeis *et al*. (1996) in which no difference in protein intake was found between overweight and non overweight children and adolescents, our findings show that percentage protein intake did not differ between quartiles of BMI z-scores or WC [18,36].

While the majority of studies have demonstrated a positive association between adiposity and dietary fat our results conflict with these findings [3,22,33,39]. An inverse relationship was observed between fat intake and BMI z-score and similar results were also found when using WC as a reference for adiposity, however these correlations were not significant. When subjects were split into quartiles based on BMI z-score and WC, there was no significant difference between the groups, which support the results found by Hassapiduo *et al*. in 2006, in which no significant difference in percentage fat intake was found in overweight and non overweight adolescents [37].

Using multiple regressions we found that the macronutrient composition of the diet had no significant impact on the ability to predict BMI z-score or WC.

Researchers have suggested that the reason why a clear relationship between total energy intake and percentage macronutrient intake and adiposity has not been seen is due to the under reporting of foods. This study applied the McCrory cut off [20] and those with an "implausible" energy intake were excluded from the analysis, thus applying a more robust method of self reported energy intake. The results suggest that the lack of association between BMI z-score and WC with macronutrient intake was not a direct result of underreporting and that total energy intake is more influential than the macronutrient composition of the diet in the development of childhood obesity.

However, perhaps one of the reasons of conflicting findings from various studies is the use of BMI itself as a measure of adiposity. While a simple, convenient assessment, the accuracy and use of the BMI as an indicator of body fatness in children is questionable [40]. Riley *et al*. 2000, suggested that BMI cut -offs are non specific, thus tending to identify non-obese "stocky" children as obese [41]. On the other hand, there is also a concern that BMI cut-offs fail to identify the obese child [40]. We attempted to partly overcome this by also exploring WC as a measure of obesity; however our correlations with WC and dietary components were very similar to those with BMI, and added nothing further to the study. It may be that WC should have been adjusted for body size. Studies investigating the relationship between dietary intake and the development of childhood obesity have used various screening tools to group children based on their level of adiposity, and as a result, the difference in methodologies between studies may account for conflicting findings.

Similarly, the use of different assessment tools to assess dietary intake in children and adolescents may explain some of the variability in previous studies assessing dietary intake and adiposity. In the current study, dietary intakes and food habits were assess by a 24 h food and drink record, similar to the 2003 Physical Activity and Nutrition Levels in Western Australian Children and Adolescents Report, which was adapted from the 1995 National Nutrition Survey. In this survey a 24 hour dietary record was used, a common approach in large population based studies. Whilst the 24 hour dietary record may not always represent habitual intake we have attempted to limit the error by screening the intake data via the method described by McCrory *et al*. [20].

## Conclusions

In light of the escalating prevalence rates of obesity and chronic disease, governments and health organisations are endeavouring to develop and implement appropriate public health strategies to prevent and manage obesity, and promote nutrition and physical activity. This study presents vital information on the energy intake, diet composition, and adiposity of Queensland children and adolescents, which may prove useful in refining policies and practice. Several studies have failed to demonstrate that overweight and obese children have a higher energy intake than healthy children. This was not the case here. We showed that total daily energy intake was higher in the overweight and obese groups. It has also been suggested that diets high in fat and low in carbohydrate may cause an accumulation of excess body fat even when total energy intake is not in excess. However, in this current study there was no evidence of such a relationship. As this was a cross -sectional study designed to provide a "snap shot" of dietary intake and anthropometry, no valid assumptions into the cause/effect relationship of obesity and dietary intake can be made and more longitudinal studies to elucidate this relationship are urgently needed, including those that use more rigorous techniques to measure body composition.

## Competing interests

The authors declare that they have no competing interests.

## Authors' contributions

SE wrote the manuscript, RA and AL critically reviewed and commented on manuscript. PSWD, HT and CH provided analytical support and commented on manuscript. All authors have read and approved the final manuscript.
